# circITGa9: A Novel Therapeutic Target for Cardiac Diseases

**DOI:** 10.34133/research.0312

**Published:** 2024-02-16

**Authors:** Xiufen Zheng

**Affiliations:** Department of Surgery, Western University, London, Ontario, Canada; Department of Pathology and Laboratory Medicine, Western University, London, Ontario, Canada; Department of Microbiology & Immunology Oncology, Western University, London, Ontario, Canada; Department of Oncology, Western University, London, Ontario, Canada; Lawson Health Research Institute, London, Ontario, Canada.

Circular RNA (circRNA) is a group of single-stranded RNAs characterized by a closed head-to-tail loop structure, produced by back-splicing of pre-mRNA post-transcription. This unique structure renders circRNAs resistant to exonuclease cleavage, resulting in a significantly prolonged half-life and increased stability compared to linear RNAs within cells. CircRNAs have emerged as crucial players in various physiological and pathophysiological processes. They play diverse roles in regulating cellular activities by functioning as robust gene regulators through interactions with microRNAs and proteins and, in some cases, direct translation into peptides/proteins and modulation of exon skipping [1].

circRNA research is an exciting and rapidly evolving field with promising prospects for future clinical application. Growing evidence has shown that circRNAs hold potential as novel biomarkers for diagnosis or therapeutic targets for treating diseases including cardiovascular diseases. It has been reported that circRNAs play critical roles in cardiac remodeling [2], repair [3], and transplantation [4]. However, circRNA research is relatively nascent, with the majority of reported studies focused on the expression of circRNA, and only limited numbers of circRNAs have been identified, characterized, and functionally evaluated. The exact molecular mechanism of a circRNA has not been completely elucidated. It is interesting and important to identify novel circRNAs, determine their effects on disease both in vitro and in vivo, and establish appropriate and robust approaches to circRNA research.

In a recent breakthrough published in *Research*, a Science Partner Journal, Li and colleagues [5] have identified a novel exonic circRNA, known as circITGa9 or has_circ_0030600, which is generated through back-splicing of exons 14 and 15 of the integrin-α9 gene, related to cardiac hypertrophy and fibrosis. The research team performed high-throughput RNA sequencing and quantitative reverse transcription polymerase chain reaction (qRT-PCR), discovering that circITGa9 expression was significantly up-regulated in ventricle tissues from patients with cardiac hypertrophy and in hypertrophic hearts from mice with transverse aortic constriction (TAC) surgery as compared with their normal heart tissues. The elevation of circITGa9 levels was also observed in human fibroblasts and cardiomyocytes cultured in vitro. circITGa9 expression adversely correlated to cardiac function, but positively correlated to collagen-I levels, which is a character of fibrosis.

Next, the authors successfully demonstrated that circITGa9 is a detrimental circRNA using a TAC murine model. The authors found that administration of circITGa9 expression plasmids increased the expression of circITGa9 in murine hearts. As a result, mice with overexpression of circITGa9 had the enlarged left ventricles, but significantly decreased heart function evidenced by reduced left ventricular ejection fraction and fractional shortening. They also found increased collagen deposition in TAC mice with overexpression of circITGa9. In the in vitro cultured primary cardiac fibroblast cells, which are major players in the development of cardiac fibrosis, the team demonstrated that overexpression of circITGa9 increased the adhesion, migration, and morphological changes of fibroblasts, activating fibrosis initiation. Taken together, the research team demonstrated that circITGa9 is a novel target for preventing cardiac hypertrophy and fibrosis.

Collectively, the authors demonstrated that the interference of circITGa9 expression could reverse the injury and protect heart function, highlighting the potential in clinic. They treated TAC mice with small interfering RNAs specifically targeting circITGa9 conjugated with gold nanoparticles to successfully silence circITGa9 in the hearts. Silencing circITGa9 not only significantly reversed the hypertrophy of left ventricle induced by TAC-mediated overload pressure but also significantly increased left ventricular pressure, averting left ventricular contraction decline. Knockdown of circITGa9 decreased levels of collagen deposition, including collagen-I and collagen-III, and reduced fibrosis. The authors proved a novel effective treatment for preventing cardiac hypertrophy and fibrosis in a murine model, showing promise of targeting circRNA therapy in clinic.

CircRNA exerts its functions through interaction with proteins [3,4]. Li and coworkers delved deeper into the mechanisms by which circITGa9 affects cardiac hypertrophy and fibrosis. Through a series of RNA immunoprecipitation, immunoprecipitation, and gain-of-function and loss-of-function assays, the authors revealed that circITGa9 binds to tropomyosin 3 (TPM3), an important protein in actin polymerization. They uncovered that the interaction of circITGa9 and TPM3 promotes actin polymerization, which in turn enhances collagen expression, resulting in fibrosis. Notably, the team creatively introduced a comprehensive computational analysis to predict the sequence of circITGa9 binding to TPM3, and then designed oligo to block the binding between circITGa9 and TPM3, to further validate the interaction, which is a novel approach for circRNA mechanism elucidation.

In summary, this groundbreaking study not only discovered a new circITGa9 involved and its vital role in cardiac hypertrophy and fibrosis through the axis of circITGa9-TPM3-actin polymerization but also verified its therapeutic significance in preventing or reversing cardiac hypertrophy and fibrosis in a murine TAC model. The study provides insights into understanding circRNA as a therapeutic target and developing targeting circRNA treatment for disease. This study also deepens our understanding of circRNA function and provides a beautiful set of methodologies for circRNA research.
